# From prescription patterns to drug safety: a closer look at non-steroidal anti-inflammatory drugs and analgesics in outpatient pharmacy

**DOI:** 10.3389/fphar.2025.1558830

**Published:** 2025-07-04

**Authors:** Zainab Mohamed Saeed, Sathvik B. Sridhar, Javedh Shareef, Amal Mohamed Rashed Alsereidi

**Affiliations:** ^1^ Department of Clinical Pharmacy and Pharmacology, RAK College of Pharmacy, RAK Medical and Health Sciences University, Ras Al-Khaimah, United Arab Emirates; ^2^ Clinical Pharmacist, Dibba Hospital, Emirates Health Services, Fujairah, United Arab Emirates

**Keywords:** drug utilization, drug interactions, outpatients, prescriptions, non-steroidal antiinflammatory agents

## Abstract

**Introduction:**

Non-steroidal anti-inflammatory medicines (NSAIDs) help to lower inflammation and pain, but improper prescription can cause potential drug-drug interactions (pDDIs), affecting the therapeutic outcomes. Given the great frequency of polypharmacy and concomitant medication interactions, NSAID-related problems are especially pertinent in outpatient settings. This study aims to assess the prescription pattern of NSAIDs at the Outpatient Pharmacy Department of a Secondary Care Hospital in the Northern Emirates of the United Arab Emirates.

**Methods:**

A prospective observational study based on data from electronic medical records of patients who received NSAID prescriptions from Jan–June 2023. Data collected were screened for prescription patterns of NSAIDs and polypharmacy, and the potential drug-drug interactions (pDDIs) were identified using the Portable Emergency Physician Information Database (PEPID). Data were extracted and analyzed using descriptive statistics and logistic regression analysis to study the association between treatment-related variables and the presence of pDDIs. Chi-square was used to test the association between the type of NSAID prescribed and co-prescribed gastro-protective agents. P < 0.05 was considered statistically significant.

**Results:**

In total, 1005 NSAID prescriptions were analyzed, with a majority (53.23%) being prescribed to female patients. Pain related to elbow/shoulder/joints/lower back (41.09%) was the most common diagnosis in the study populations. Celecoxib (49.7%) was the most commonly prescribed oral, and Ketoprofen (39.5%) was the predominant topical NSAID. A significant association was found between the prescribed NSAID and its co-prescription with gastroprotective agents, specifically ibuprofen, celecoxib, piroxicam, meloxicam (P < 0.001), diclofenac (P = 0.007), and aspirin (P = 0.001). Age and chronic illnesses such as diabetes mellitus (OR = 1.446; 95% CI: 1.018–2.054) and cardiovascular disease (OR = 1.818; 95% CI: 1.279–2.585) are significantly associated with polypharmacy. Multiple logistic regression analysis revealed that the pDDIs were significantly higher with an increasing number of prescribed drugs and co-morbidities (p < 0.001).

**Conclusion:**

The study examines NSAID prescribing trends and emphasizes the potential for drug-related issues, particularly in light of polypharmacy, which calls for careful monitoring and prescribing practices. Healthcare providers should routinely conduct medication reviews and team up with clinical pharmacists to ensure rational NSAID use, reduce drug interactions, and enhance patient safety by thus mitigating this risks.

## Introduction

A basic component of outpatient treatment, especially in secondary care environments where analgesics and non-steroidal anti-inflammatory medications (NSAIDs) are routinely provided, is effective pain management (Fenske et al., 2021). Widely used to treat musculoskeletal problems, headaches, visceral discomfort, surgical pain, and acute or chronic arthritic disorders, common recommended NSAIDs include ibuprofen, naproxen, and diclofenac (Ghlichloo and Gerriets, 2024). Their natural importance in improving patient quality of life thus emphasizes their efficiency in reducing pain and inflammation, so securing their position in clinical practice (Magni et al., 2021).

Although NSAIDs are clinically useful, data indicates that they are likely to have side effects including high blood pressure, cardiovascular disease, kidney damage, and gastrointestinal (GI) problems including ulcers and bleeding (Bindu et al., 2020). Particularly for the high-risk groups, these negative effects highlight careful prescribing and close monitoring. Usually, the doctors in clinical practice co-prescribes acid suppressive drugs to reduce these dangers (Tai and McAlindon, 2021). Optimizing patient care and certifying thorough treatment depend on knowing the regular patterns about the use of acid suppressive drugs in line with NSAIDs as well as the reasons behind these practices. Simultaneously, it is rather important to strengthen clinical safety by closely examining prescribing practices (Sohail et al., 2023).

Globally, considerable variation in NSAID prescribing practices has been noted in the class and types of NSAIDs used, their dosages, modes of administration, and therapy length. This erraticism can result in different treatment results and a higher risk of negative health effects (Abdu et al., 2020). World Health Organization supports comparison of Prescribed Daily Doses (PDD) to Defined Daily Doses (DDD) in order to evaluate the adherence with the safe prescribing guidelines (Masclee et al., 2018). Variations between PDD and DDD could highlight improper dosing methods that might compromise the efficacy of the treatment or patient safety (Nunes et al., 2022).

In pharmacotherapy, drug interactions are a prominent issue that especially affects NSAIDs when co-prescribed with other drugs. It is well known that concurrently when used with anticoagulants increases risk of hemorrhage whereas, with renin angiotensin pathway inhibitors and diuretics may cause renal impairment (Bindu et al., 2020). Evaluating the prevalence, severity, and level of concern regarding drug-drug interactions among NSAID users is essential for improving treatment regimens and safeguarding patient health (Hamadouk et al., 2023).

The incidence of polypharmacy among NSAID users and the associated risk factors warrants investigation to understand its impact on patient outcomes and healthcare costs (National Institute of Aging, 2021). Polypharmacy can complicate treatment regimens and raise the risk of harmful drug interactions, emphasizing the importance of proper medication management (Abdu et al., 2020; Varghese et al., 2024).

In the context of the United Arab Emirates (UAE), NSAIDs are some of the most widely prescribed medications in outpatient settings, particularly for musculoskeletal and inflammatory conditions (Hassan et al., 2021; Alomar et al., 2019). This high prevalence highlights the need for a detailed understanding of NSAID prescription patterns to ensure that therapeutic outcomes are optimized and potential adverse effects are minimized. The UAE’s unique healthcare landscape, influenced by local clinical guidelines, cultural practices, and the availability of medications, can significantly impact NSAID prescribing behaviors (Department of Health Abu Dhabi, 2020). Therefore, examining these factors within the UAE context is crucial for developing appropriate and effective pain management strategies.

Understanding NSAID prescription patterns in the UAE is crucial for improving clinical practices and patient outcomes. This study will contribute to the broader body of knowledge on NSAID use, offering evidence-based recommendations for optimizing pain management and reducing the risks associated with NSAID therapy. This research aims to address these gaps in knowledge by assessing the prescription patterns of NSAIDs at the outpatient pharmacy department of a secondary care hospital in the UAE. The study will evaluate commonly prescribed NSAIDs, including their types, dosages, routes of administration, and duration of therapy. It will also analyze the use of gastro protective agents co-prescribed with NSAIDs, compare PDD with DDD to assess adherence to dosage guidelines and investigate potential drug-drug interactions (pDDIs) and the incidence of polypharmacy among NSAID users. By achieving these objectives, the research seeks to provide valuable insights into current prescribing practices, enhance patient safety, and inform future healthcare strategies. Ultimately, the findings will support efforts to refine prescribing practices, enhance patient safety, and ensure patients receive effective and appropriate pain management in outpatient settings.

## Methodology

### Study design and setting

A prospective observational study was carried out on all patients visiting the outpatient pharmacy department for 6 months (January 203–June 2023) in the Dibba Hospital, Al-Fujairah, U.A.E.

### Sample size

The Epi Info™ software from the CDC (version 7.2) was used to determine the required sample size (Centers for Disease Control, 2024). The calculation was based on a 5% margin of error, a 95% confidence level, and a 50% response distribution, resulting in a required sample size of approximately 1,000 patients.

This sample size was chosen not only for its statistical reliability but also to ensure adequate representation of different NSAID types, comorbid conditions, and potential drug interactions over the 6-month period. Additionally, it was feasible within the resource and time constraints of the study conducted in a single secondary care public hospital.

### Sampling technique

A systematic random sampling technique was used to select patient prescriptions. The sampling was conducted over a 6-month period. A list of all outpatient prescriptions containing NSAIDs was extracted from the hospital’s pharmacy database. From this list, every *k*th prescription was selected, where k was determined by dividing the total number of eligible NSAID prescriptions during the study period by the required sample size (1,000). The first prescription was selected at random within the first k interval to ensure randomness. Each selected prescription corresponded to one unique patient.

### Criteria for inclusion and exclusion

All male and female patients aged 18 years or older who were prescribed at least one systemic NSAID along with at least one additional medication in the outpatient departments of the study hospital were included. Informed consent was obtained from all participants.

Patients were excluded if they were admitted to inpatient wards, the intensive care unit (ICU), or the emergency department, or if they were prescribed only topical creams, ointments, or compounded items for which complete prescription data were unavailable or inadequately recorded.

### Study procedure

All patient prescription records that satisfied the inclusion criteria were incorporated into the study. A structured data collection form was used to systematically extract key variables that captured patient demographics (age, gender, nationality), clinical details (diagnoses and comorbidities), and prescription information. NSAID-specific variables included the type of NSAID prescribed, route of administration, dose, frequency, duration, co-prescribed medications (including gastroprotective agents), and the number of drugs per prescription for polypharmacy and pDDI assessment from the electronic health records (Wareed system) at the outpatient pharmacy department of the study setting. Wareed system, an electronic health information system (HIS) developed by the Ministry of Health and Prevention (MOHAP) in the UAE. It integrates all MOHAP-run medical facilities to enable standardized, paperless, and efficient healthcare delivery. The system captures patient demographics, clinical diagnoses, and prescribed medications, supporting comprehensive retrieval of outpatient prescription records. As with all EMRs, the accuracy of the data depends on proper documentation by healthcare professionals.

Prescriptions for NSAIDs were collected prospectively and screened to assess prescribing patterns. Each prescription containing NSAIDs was evaluated according to specified study parameters (type, brand name, dose, administration route, therapy duration, monotherapy fixed drug combination, etc.).

Medications were categorized according to the WHO Anatomical Therapeutic Chemical (ATC) classification system. Prescription data from commonly prescribed drug categories was used to convert consumption into defined daily doses (DDD) based on the 2022 ATC/DDD index. The DDD was calculated by multiplying the number of items dispensed by the drug amount per item and dividing by the WHO DDD metric. The number of DDDs per 100 bed days was then determined. Finally, the estimated Prescribed Daily Dose (PDD) in grams was calculated by multiplying the DDD by the ratio of DDDs to treatment days (World health Organization., 2023).

Medications were categorized using the WHO Anatomical Therapeutic Chemical (ATC) classification system. Prescription data from commonly prescribed drug categories were used to convert consumption into Defined Daily Doses (DDD) based on the 2022 ATC/DDD index.

To calculate the DDD, the following method was used.

• Number of items dispensed: The total number of medication units (e.g., tablets, capsules) dispensed to the patient during the study period was recorded.
• Drug amount per item: The amount of the active ingredient in each unit of the medication was noted (e.g., mg per tablet).
• Conversion to DDD: The DDD value for each medication is defined by WHO as the assumed average maintenance dose for an adult in a standard treatment regimen. To convert the total number of items dispensed into DDDs, the following formula was applied:

$$\text{DDD}=\frac{\left(\text{Number}\;\text{of}\;\text{items}\;\text{dispensed}\right)\times \left(\text{Amount}\;\text{of}\;\text{drug}\;\text{per}\;\text{item}\right)}{\text{DDD}\;\text{from}\;\text{the}\;\text{WHO}\;\text{index}\left(\text{for}\;\text{that}\;\text{specific}\;\text{drug}\right)}$$

This calculated value was then used to determine the number of DDDs per 100 bed days.

Finally, the Prescribed Daily Dose (PDD) in grams was calculated by multiplying the DDD by the ratio of DDDs to treatment days (Norwegian Institute of Public Health., 2022).

### Assessment of polypharmacy

In this study, polypharmacy was assessed by analyzing the number of medications prescribed to patients at the outpatient pharmacy department. Given that there is no universally agreed-upon definition of polypharmacy, we followed a widely accepted classification used in various studies (Lizhen et al., 2022; Pazan and Wehling, 2021), which categorizes polypharmacy into the following levels: non-polypharmacy (fewer than five), polypharmacy (five to nine), and hyper polypharmacy (ten or more medications). However, it is important to note that some studies have defined polypharmacy as three or more medications, but this threshold is less commonly used in clinical settings compared to the five-medication threshold.

### Assessment of pDDIs

To recognize possible drug-drug interactions (pDDIs) relating to NSAID prescriptions, every medication prescribed, apart from those specified as ‘stat’ or ‘as needed,’ was incorporated into the ‘drugs to check’ list. This list was then analyzed using the clinical decision support system, Portable Emergency and Primary Care Information Database (PEPID), to screen for possible DDIs.

### Mechanism of interaction

Medication interactions were classified into pharmacokinetic (PK), pharmacodynamic (PD), or other mechanisms.

#### Severity level of the pDDIs

Interactions in PEPID are depicted by colored warning triangles, indicating severity from highest to lowest. Each triangle includes a number corresponding to the severity level. A “5″denotes a life-threatening interaction that should never be used. Level 4 indicates a serious interaction with a high risk of severe consequences, only to be considered if the benefits outweigh the risks. Level 3 requires close monitoring and consideration of alternatives. Level 2 also demands careful monitoring, while Level 1 signifies a minor interaction. For the purposes of this study, only level 1 (non-significant) interactions were excluded from further analysis. Levels 2 through 5 were included and analyzed as minor, moderate, significant and life-threatening respectively.

### Data analysis

Data were analyzed using IBM SPSS Statistics version 28. Descriptive statistics were used to summarize demographic, clinical, and prescribing characteristics. Chi-square tests and Relative Risk (RR) calculations were used to assess associations between categorical variables such as polypharmacy, comorbidities, and the presence of pDDIs, as these tests are appropriate for comparing proportions across groups. Pearson correlation analysis was employed to explore the strength and direction of relationships between continuous variables (e.g., number of prescribed drugs and number of pDDIs).

Univariate and multivariate logistic regression analyses were conducted to identify independent predictors of potential drug-drug interactions (pDDIs), as logistic regression is suitable for modeling the probability of a binary outcome (presence or absence of pDDIs) based on multiple predictors. Potential confounding variables such as age, gender, comorbidities (e.g., diabetes, cardiovascular disease), and the number of prescribed medications were selected based on clinical relevance and findings from previous literature (Rasool et al., 2023; Sah et al., 2023; Gobezie et al., 2021). These variables were included in the multivariable logistic regression analysis to control for their potential confounding effects on the outcomes. A p-value of <0.05 was considered statistically significant, and p < 0.01 indicated a highly significant association.

## Results

### Demographic details of the study populations

One thousand-five patients each contributing one outpatient prescription were included in the study from the outpatient pharmacy department. Among them, 535 (53.23%) were female, primarily receiving NSAID prescriptions (509, 50.64%), and aged 26 to 50, with 718 (71.4%) being UAE nationals. The average age of the patients was 46.90 ± 16.29 years, ranging from 18 to 103 years. More than 90% of the study populations were non-alcoholics and non-smokers and reported that they did not have any previous allergy history. Considering their occupational status, most patients were unemployed, 620 (61.69%), and the majority (744, 74.02%) were married, as reported in their marital status. The analysis of prescriptions revealed an average of 4.66 ± 2.69 drugs per patient, with a range of 1–18. Most study patients [736 (73.23%)] received 1-5 drugs (Table 1).

**TABLE 1:** Socio-demographic characteristics of the study population.

Variable	N = 1,005 (%)	95% confidence interval
Gender		
Female	535 (53.23)	50.0–56.6
Male	470 (46.8)	43.4–50.0
Age (In Years)		
≤25	92 (9.15)	7.3–10.9
26–50	509 (50.64)	47.6–54.0
51–75	364 (36.21)	33.3–39.4
>75	40 (3.98)	2.9–5.3
Nationality		
UAE Nationals	718 (71.40)	68.7–74.0
Expatriates	287 (28.55)	26.0–31.3
Employment Status		
Employed	351 (34.92)	32.1–37.8
Not Employed	620 (61.69)	58.7–64.6
Retired	34 (3.38)	2.3–4.6
Alcohol use		
Yes	07 (0.69)	0.3–1.3
No	998 (99.30)	98.7–99.7
Smoking		
Yes	44 (4.37)	3.1–5.8
No	961 (95.62)	94.2–96.9
Marital status		
Single	220 (21.89)	19.3–24.4
Married	744 (74.02)	71.3–76.8
Widowed	34 (3.38)	2.3–4.5
Divorced	07 (0.69)	0.2–1.3
Number of comorbidities		
Nil	474 (47.16)	44.2–50.3
1–2	416 (41. 39)	36.3–46.5
3–4	108 (10.74)	8.1–13.6
≥ 5	07 (0.68)	0.2–1.4
Total No. of Prescription Drugs		
1–5	736 (73.6)	70.3–75.8
6–10	216 (21.6)	19.2–24.2
11–15	43 (4.3)	3.1–5.5
>16	10 (1.0)	0.5–1.6

### Number of comorbidities

**FIGURE 1:**
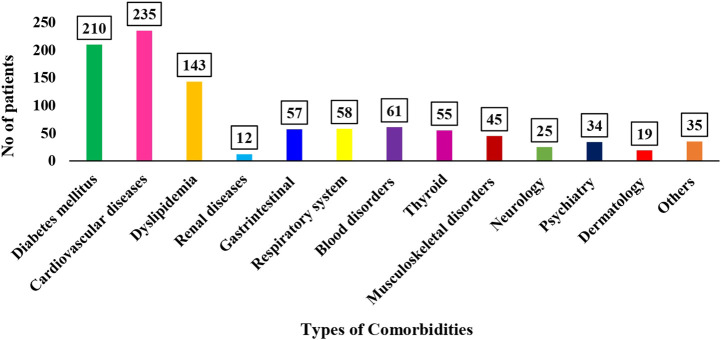
Types of different comorbidities present among study populations.

A total of 983 comorbid conditions were recognized within the study groups. It was observed that more than half (52.8%) had one or more comorbidities, with disease related to cardiovascular [235 (23.9%)] being the most common (Figure 1).

### Nature of different prescribed NSAIDs dosage forms among study patients

Among the total 1,005 prescriptions analyzed, a total of 2051 NSAIDs were prescribed. Most of the study patients [351 (34.9%)] received two NSAID drugs per prescription, followed by 320 (31.8%) patients who received three NSAID drugs per prescription. It was observed that 573 (57. 01%) of the patients received topical and oral NSAID medications per prescription. However, 411 (40.89%) patients received oral NSAIDs, and 21 (2.08%) patients were prescribed only topical NSAIDs. At the same time, the fixed dosage regimen, such as paracetamol with Orphenadrine citrate, was prescribed to 253 (25.17%) of the patients, and paracetamol with tramadol was received by one patient (0.09).

### Current medical diagnosis of the study patients

In our study, the majority (423, 34.9%) of the patients included were diagnosed with acute or chronic pain related to shoulder/elbow/joint/low back aches and body aches followed by spondylosis/radiculopathy-related pain (259, 21.4%), arthritis (143, 11.81%) and dental caries/tooth decay (104, 8.59%) (Table 2).

**TABLE 2:** Types of diagnosis among the study patients.

Sl No.	Diagnosis	Frequency	%
1	Pain in shoulder/joint/elbow/low back/body ache	423	34.95
2	Spondylosis/radiculopathy	259	21.4
3	Arthritis related pain	143	11.81
4	Dental/tooth/gingivitis	104	8.59
5	Others (Otalgia, tonsillitis, lymphadenitis, foot trauma, kidney stone)	71	5.86
6	Bilateral plantar fasciitis/fracture	68	5.61
7	Pain unspecified disease/disorder	65	5.37
8	Chronic diseases related pain (gout, inflammatory bowel disease, diabetes mellitus, iron deficiency anemia)	43	3.55
9	Osteoporosis/hemorrhoids	23	1.9
10	Neuropathic pain	11	0.9

### Prescribing pattern of NSAID medications according to therapeutic class of medications

In this study, NSAID prescriptions were categorized by pharmacologic class. Among analgesic/antipyretic drugs with poor anti-inflammatory action, paracetamol (N02BE01) was prescribed in 45.4% of cases (n = 456, CI: 42.2–48.6). Non-selective COX inhibitors included ketoprofen (39.5%), ibuprofen (16.3%), piroxicam (10.8%), indomethacin (2.5%), and aspirin (3.1%). Preferential COX-2 inhibitors were diclofenac (21.6%) and meloxicam (14.7%). Celecoxib was the only selective COX-2 inhibitor used and accounted for 49.7% of NSAID prescriptions. These data indicate a high preference for celecoxib among selective agents and ketoprofen among topical formulations (Table 3).

**TABLE 3:** Therapeutic class of NSAID medications with individual drugs.

NSAID Category	Drugs Name	ATC Code	n	%	CI 95%
⁃ Analgesic/Antipyretic with poor anti-inflammatory action	⁃ Paracetamol	N02BE01	456	45.4	42.2–48.6
⁃ Non-selective COX inhibitors	⁃ Aspirin	B01AC06	31	3.1	2.1–4.2
	⁃ Ibuprofen	M01AE01	164	16.3	14.3–18.7
	⁃ Indomethacin	M02AA23	25	2.5	1.6–3.5
	⁃ Ketoprofen	M02AA10	399	39.5	36.6–42.7
	⁃ Piroxicam	M01AC01	109	10.8	9.0–12.7
⁃ Preferential COX-2 Inhibitors	⁃ Diclofenac	M01AB05	220	21.6	18.8–24.6
	⁃ Meloxicam	M01AC06	148	14.7	12.5–17.1
⁃ Selective COX-2 inhibitors	⁃ Celecoxib	M01AH01	499	49.7	46.4–52.7

### Frequency of NSAID prescriptions based on patient age and gender

The analysis of NSAID prescribing frequencies by gender and age shows significant differences for several drugs. Aspirin, Ibuprofen, Ketoprofen, Piroxicam, and Paracetamol strongly correlate with age and gender (p-values <0.05), indicating varying prescription patterns across these factors. For example, higher prescriptions of Ketoprofen and Ibuprofen are seen in older age groups. Celecoxib also shows a gender-age interaction, while no significant gender-age differences were observed for Diclofenac, Indomethacin, and Meloxicam (p-values >0.05).

### The relationship between the type of NSAID and the co-prescription of a gastroprotective agent

A total of 424 patients (42.2%) were co-prescribed proton pump inhibitors (PPIs) alongside NSAIDs. Among the PPIs, omeprazole was the most frequently prescribed (50.4%), followed by dexlansoprazole (26.6%), esomeprazole (17.4%), and pantoprazole (4.0%). Analysis of the relationship between the type of NSAID and the co-prescription of a gastroprotective agent revealed statistically significant associations for celecoxib (48.9%, p < 0.0001), meloxicam (59.5%, p < 0.0001), aspirin (70.9%, p = 0.001), diclofenac (35.7%, p = 0.007), and ibuprofen (23.8%, p < 0.0001). These results suggest that PPIs, particularly omeprazole, are commonly used to prevent gastrointestinal complications in patients prescribed NSAIDs with known or perceived higher GI risk (Table 4).

**TABLE 4:** Association between types of NSAIDs prescribed and whether it is co-prescribed with a gastroprotective agent (s).

Type of NSAIDs	Co-prescription with gastro-protective agents			P-value
	Yes	No	n	
Ibuprofen	39 (23.78)	125 (76.21)	164	<0.0001
Celecoxib	244 (48.89)	255 (51.10)	499	<0.0001
Diclofenac	79 (35.74)	141 (63.80)	220	0.007
Piroxicam	28 (25.68)	81 (74.31)	109	<0.0001
Ketoprofen	167 (42.06)	230 (57.93)	399	0.944
Paracetamol	193 (42.51)	263 (57.48)	456	0.887
Aspirin	22 (70.96)	9 (29.03)	31	0.001
Indomethacin	14 (56)	11 (44)	25	0.151
Meloxicam	88 (59.45)	60 (40.54)	148	<0.0001

p ≤ 0.05 was considered significant.

### PDD, DDD, and PDD/DDD ratio for the NSAID medications

The PDD, calculated by dividing the total dose by the number of days, showed a ratio of 1 for Piroxicam and diclofenac, while paracetamol, ibuprofen, and meloxicam had ratios below one (Table 5).

**TABLE 5:** PDD, DDD, and PDD/DDD ratio for the prescribed NSAIDs.

Type of NSAID medications	Route of administration	No. of prescriptions (%)	ATC code	PDD	DDD	PDD/DDD
Analgesic/Antipyretics						
• Paracetamol	Oral	456	N02BE01	1.68	3 gm	0.56
Non-selective COX inhibitors						
• Aspirin	Oral	31	B01AC06	1.57	1	1.57
• Ibuprofen	Oral	164	M01AE01	1.19	1.2	0.99
• Indomethacin	Topical	25	M02AA23	—	—	
• Ketoprofen	Topical	397	M02AA10	—	—	
• Piroxicam	Oral	109	M01AC01	20	20 mg	1.0
Preferential COX-2 Inhibitors						
• Diclofenac	Oral	47	M01AB05	0.1	0.1 gm	1.0
	Topical	173	M02AA15		—	
• Meloxicam	Oral	148	M01AC06	14.4	15 mg	0.94
Selective COX-2 inhibitors						
• Celecoxib	Oral	499	M01AH01	0.25	0.2 gm	1.25

### Incidence of polypharmacy among NSAID users and associated risk factors

Of the 1,005 patients, 383 (38.41%) received five or more drugs per prescription, categorizing them as major polypharmacy. Age and chronic illnesses such as diabetes and cardiovascular disease are significantly associated with polypharmacy. Individuals aged 31–60 years had significantly lower odds of polypharmacy compared to those under 30 years (OR = 0.254; 95% CI: 0.148–0.434; p < 0.001), and those over 60 also had lower odds (OR = 0.598; 95% CI: 0.423–0.845; p = 0.004). Although statistically significant, the clinical relevance of these odds ratios should be interpreted with caution due to their small effect sizes. Patients with diabetes mellitus (OR = 1.446, 95% CI: 1.018–2.054), and individuals with cardiovascular diseases (OR = 1.818, 95% CI: 1.279–2.585) were more likely to be exposed to polypharmacy. This suggests that the presence of certain chronic diseases, particularly cardiovascular and metabolic conditions, may be predictive factors for polypharmacy (Table 6).

**TABLE 6:** Association of Polypharmacy with Demographic and Clinical Factors by using bivariate Analysis.

Variables	Bivariate analysis	
	Crude OR (95%CI)	p-value
Age		
Less than 30	Ref	
31–60	0.254 (0.148–0.434)	**0.0001**
More than 60	0.598 (0.423–0.845)	**0.004**
Gender		
Male	Ref	
Female	0.846 (0.645–1.11)	0.229
Diabetes mellitus		
Yes	1.446 (1.018–2.054)	**0.040**
No	Ref	
Cardiovascular system		
Yes	1.818 (1.279–2.585)	**0.001**
No	Ref	
Chronic kidney disease		
Yes	3.337 (0.845–13.18)	0.086
No	Ref	
Rheumatology		
Yes	1.891 (0.990–3.613)	0.054
No	Ref	
Respiratory		
Yes	1.414 (0.810–2.468)	0.222
No	Ref	

Ref = Reference category for odds ratio (OR) calculation. Bold values indicates that p < 0.05 statistically significant; p < 0.01 statistically highly significant.

### Prevalence of pDDIs

pDDIs were documented in 731 out of 1,005 study patients, resulting in a prevalence of 72.73% among the participants. An analysis using the PEPID database interaction checker identified 1,251 pDDIs and 45 pairs of interacting drugs among the 1,005 patients. However, 88 pDDIs were deemed non-significant and excluded from further analysis. The majority of the patients [519 (51.64%)] had 1-2 pDDIs per prescription, followed by 88 (8.75%) patients who were found to have 3-4 pDDIs per prescription and 27 (2.68%) patients who had 5-6 pDDIs in their prescriptions. There were 9 (0.89%) patients having seven or more pDDIs in their prescriptions and 362 (36.01%) patients had no pDDIs in their prescriptions.

### Severity of pDDIs

Among the 1,163 pDDIs identified from 731 study patients, fourteen pairs of pDDIs were categorized as significant (1.20%), 434 (37.3%) were moderate in severity, and 715 pDDIs belonged to the category ‘mild’ in level of severity. There were no pDDis belonging to the category as life-threatening (level 5) were detected in this study.

### Type of pDDIs

Out of the total 1,163 pDDIs identified in study patients, 600 pDDIs (51.59%) were pharmacodynamics type interactions, while 320 (27.51%) were pharmacokinetic drug interactions. More than half of the pDDIs (600, 51.59%) were rated as ‘other mechanisms’ involved in causing pDDIs. Considering the ‘source of recommendations’ of pDDIs, 753 (64.73%) pDDIs were predicted and recognized based on pharmacokinetic or pharmacodynamic principles, while 241 (20.72%) interactions were of the drug interactions available in the literature and 161 (16.81%) interactions were predicted drug/drug interaction based on pharmacokinetic or pharmacodynamic principles.

### Types of pDDIs

Forty-five pairs of interacting drugs associated with different medicines in the study patients were identified. The most common types of pDDIs were celecoxib + Omeprazole (244,20.98%) followed by celecoxib + Orphenadrine (131, 11.26%) and celecoxib + sodium aesinate (129,11.09%) (Table 7).

**TABLE 7:** Frequency and Nature of pDDIs in NSAIDs Outpatients identified in the study populations.

S. No	Drug pairs	N (%)	Severity	MOA	Mechanism of interaction & effect	SOR
1	Celecoxib + Warfarin	01 (0.08)	Significant	PD	Both increase anticoagulation Increased risk of bleeding	pa
2	Celecoxib + Escitalopram	02 (0.16)	Significant	Other	Increased risk of upper GI bleeding. SSRIs inhibit serotonin uptake by platelets	a
3	Celecoxib + Orphenadrine	131 (11.26)	Moderate	PD	Both decrease cholinergic effects/transmission and Increased risk of anticholinergic syndrome	p
4	Celecoxib by omeprazole	244 (20.98)	Minor	PK	Celecoxib levels can be increased, Increased risk of nausea, vomiting, abdominal pain	pa
5	Piroxicam + Valsartan	09 (0.77)	Moderate	PD	Both increase serum potassium	pa
6	Piroxicam + Chondroitin/glucosamine	17 (1.46)	Minor	PD	Both increase anticoagulation Increased risk of bleeding	P
7	Aspirin + Escitalopram	01 (0.08)	Significant	Other	Increased risk of upper GI bleeding. SSRIs inhibit serotonin uptake by platelets	a
8	Aspirin + Lisinopril	04 (0.32)	Moderate	Other	Effects may be decreased. The combination also has the potential for compromised renal function, including acute renal failure	a
9	Aspirin + Meloxicam	05 (0.42)	Moderate	PD	Both increase serum potassium	pa
10	Aspirin + Bisoprolol	05 (0.42)	Moderate	PD	Both increase serum potassium	pa
11	Aspirin + Gliclazide	08 (0.68)	Minor	Other	Risk of hypoglycemia	a
12	Ibuprofen + Escitalopram	01 (0.08)	Significant	Other	Toxicity may be increased; Increased risk of upper GI bleeding. SSRIs inhibit serotonin uptake by platelets	a
13	Ibuprofen + Apixaban	01 (0.08)	Significant	PD	Both increase anticoagulation Increased risk of bleeding	p
14	Ibuprofen + Bisoprolol	02 (0.16)	Moderate	PD	Both increase serum potassium	pa
15	Ibuprofen + Omeprazole	39 (3.35)	Minor	PK	Ibuprofen levels can be increased Increased risk of GI, renal, and hepatic toxicity	pa
16	Ibuprofen + Amoxicillin	17 (1.46)	Minor	Other	Both have similar mechanisms of decreasing renal clearance; Both levels may be increased	a
17	Meloxicam + Eplerenone	01 (0.08)	Significant	PD	Both increase serum potassium	pa
18	Meloxicam + Indapamide	05 (0.42)	Moderate	PD	One drug increases and the other drug decreases the PDI Mechanism, so the net effect is not clear	p
19	Diclofenac + Amoxicillin	13 (1.11)	Minor	Other	Both have similar mechanisms of plasma protein binding competition; Both levels may be increased	a

LOC, level of concern; MOA, mechanism of action; Source of Recommendation (SOR).

p, Predicted drug/drug interaction based on pharmacokinetic or pharmacodynamic principles; a, drug/drug interaction in literature; pa, predicted and recognized drug/drug interaction based on pharmacokinetic or pharmacodynamic principles.

### Association of demographic, disease, and treatment variables with pDDI presence

A significant association (P < 0.05) between potentially dangerous drug-drug interactions (pDDIs) and factors such as gender (X^2^ = 5.73; p = 0.017), nationality (X^2^ = 8.188; p = 0.005), and comorbidities (X^2^ = 4.66; p = 0.031). There was also a strong correlation (p < 0.01) between the total number of drugs prescribed (X^2^ = 22.958; p < 0.0001) and age groups (X^2^ = 17.23; p < 0.001).

The calculation of relative risk to identify the predictors of potential DDIs reveals that the total number of drugs prescribed [(p=< 0.0001; RR = 95% CI = 0.126 (0.056–0.282] and presence of comorbidities [(p = 0.033; RR = 95% CI = 0.860 (0.747–0.991] were the predictor of pDDIs. However, variables such as age did not show any statistically significant association with the presence of pDDIs.

### Correlation between number of drug-drug interactions and treatment-related variables

The Pearson correlation test shows a significant positive correlation between the number of potential drug-drug interactions (pDDIs) and several factors: gender (r = 0.063; p = 0.044), age (r = 0.254; p < 0.001), number of comorbidities (r = 0.201; p < 0.001), and the total number of medications received (r = 0.650; p < 0.001). However, with gender, the correlation is weak (r < 0.1), indicating that while statistically significant, the clinical association is minimal.

### Predictors of pDDIs

The multiple logistic regression showed that age [(p = 0.026; OR = 0.988; 95% CI = 0.988 (0.977–0.999] and the total number of prescribed medications [(p= <0.0001; OR = 95% CI = 1.685 (1.523–1.861] were the only factors that significantly predicted pDDIs. Despite achieving statistical significance, the effect size of age factor is very small (OR close to 1), suggesting limited clinical importance.

## Discussion

NSAIDs are frequently prescribed for pain management, but their use is accompanied by significant risks, particularly concerning gastrointestinal and cardiovascular complications (Tai and McAlindon, 2021). This study aimed to assess NSAID prescribing patterns, the prevalence of potential drug-drug interactions (pDDIs), polypharmacy, and the association of comorbidities with NSAID use. The following discussion delves into the key findings, their clinical implications, and offers actionable recommendations for improving NSAID prescribing practices.

We observed nearly equal NSAID prescribing between genders (53.23% versus 46.76%), contrasting with studies reporting higher use in women (Nasution and Mukhtar, 2019; Alshakka et al., 2018). This could reflect differences in healthcare access, gender-specific pain reporting, or regional prescribing patterns, as cultural and health-seeking behaviors can influence NSAID prescribing trends. Recognizing gender differences is essential in pain management, as it can inform more targeted prescribing strategies.

Regarding age, the majority of NSAID prescriptions were for adults aged 25–50 years aligning with previous studies (Meng et al., 2021; Wongrakpanich et al., 2018). However, discrepancies with studies showing higher NSAID use in older adults may be due to regional healthcare practices, prescribing behaviors, and the increased prevalence of musculoskeletal conditions in older populations, which are more prone to NSAID use. Additionally, younger adults may be more likely to seek outpatient care for musculoskeletal complaints or injuries, leading to higher NSAID prescriptions in this group.

Moreover, a significant proportion of the study population had comorbidities, with cardiovascular disease being the most prevalent, followed by diabetes mellitus. This finding shadows the previous studies with similar trends in their populations (Meng et al., 2021; Al-Tale and Albasry, 2021; Chaudhari and Chaudhary, 2023). Although our study did not capture clinical outcomes, the high prevalence of NSAID use and pDDIs in patients with cardiovascular conditions suggests a substantial risk for adverse events such as myocardial infarction, heart failure exacerbation, or hospitalization. Studies have shown that even short-term NSAID use in high-risk populations can elevate cardiovascular events (Sondergaard et al., 2017). These findings highlight the need for prescribers to weigh the benefits of NSAID therapy against long-term cardiovascular risks and consider safer alternatives or enhanced monitoring strategies in such patients (Forbes et al., 2024; Lin et al., 2022).

Pain related to musculoskeletal issues such as shoulder, joint, elbow, and lower back pain was the main clinical reason for NSAID prescriptions in our study. Other indications included spondylosis, arthritis, fractures, osteoporosis, and neuropathic pain. This finding aligns with preceding studies that identified musculoskeletal pain as a common reason for NSAID use (Anandan et al., 2019; Kandasamy et al., 2021).

Non-selective COX inhibitors were the most frequently prescribed NSAIDs, particularly oral ibuprofen (16.31%) and topical ketoprofen (39.5%). The increased use of gel may be due to its ability to relieve symptoms while minimizing systemic side effects. This high frequency prescription patterns mirror with other hospital-based studies (Meng et al., 2021; Ide et al., 2024; Yang et al., 2023; Zeinali et al., 2017). However, despite the established efficacy of NSAIDs, improper use can lead to significant safety issues and drug-related morbidity.

Non-selective COX inhibitors, especially oral ibuprofen (16.31%) and topical ketoprofen (39.5%), were the most commonly prescribed NSAIDs in our cohort. This aligns with other studies conducted in similar hospital settings (Meng et al., 2021; Ide et al., 2024; Yang et al., 2023). Interestingly, selective COX-2 inhibitors like celecoxib were also prescribed, which have been associated with a better safety profile, particularly in terms of gastrointestinal risks (Sohail et al., 2023; Shin, 2018). The PRECISION trial demonstrated that selective COX-2 inhibitors are associated with reduced risks of gastrointestinal problems and cardiovascular incidents in comparison to non-selective NSAIDs like ibuprofen and naproxen (Reed and Nissen, 2019).

Omeprazole was the most commonly prescribed proton pump inhibitor (PPI) in our study, reflecting clinical awareness of the gastrointestinal (GI) risks associated with NSAID use. Its dominant use may be attributed to its widespread availability, affordability, and endorsement in clinical guidelines for preventing NSAID-induced GI complications.

We observed significant associations between PPI co-prescription and specific NSAIDs such as celecoxib, meloxicam, aspirin, and diclofenac. These agents are either linked to higher GI toxicity or are frequently prescribed to high-risk patient groups. Interestingly, even selective COX-2 inhibitors like celecoxib and meloxicam—designed to reduce GI side effects—were often co-prescribed with PPIs. This likely reflects a cautious approach by clinicians, especially when managing elderly patients or those with comorbidities.

Despite guideline recommendations supporting gastroprotection for at-risk NSAID users, only 42.18% of patients in our study were co-prescribed PPIs. This rate is lower than reported in other settings, such as the UK and Canada, where co-prescription rates range from 50% to over 70% among high-risk groups (Wilcox et al., 2008; Targownik et al., 2010). Possible reasons for this underutilization include limited prescriber awareness, variability in guideline adherence, or underestimation of patient-specific GI risk factors such as age, comorbidities, and concurrent medications.

These findings underscore a critical gap in preventive care and highlight the need for improved implementation of gastroprotective strategies. Although our study documented PPI co-prescription, it did not assess whether these decisions aligned with clinical guidelines based on individual risk factors. Future research should address the appropriateness of gastroprotection in NSAID users to promote safer and more effective pain management.

### Defined daily dose (DDD) analysis

The DDD methodology was used to evaluate NSAID consumption patterns by comparing the prescribed daily dose (PDD) to the defined daily dose (DDD) set by WHO. Previous studies have indicated significant variations between PDD and DDD for many NSAID medications (Nelson et al., 2019; Bakrin et al., 2020). Our data showed that for some NSAIDs, such as aspirin (PDD/DDD = 1.57) and celecoxib (1.25), the PDD exceeded the DDD. This could reflect clinical decisions to use higher doses for specific indications such as chronic inflammatory conditions or cardiovascular protection (in the case of aspirin). For others, like ibuprofen (0.99), diclofenac (1.0), and piroxicam (1.0), PDDs were closely aligned with or equal to their DDDs, suggesting standardized dosing practices. On the other hand, meloxicam (0.94) and paracetamol (0.56) showed lower PDDs compared to their DDDs, possibly due to cautious prescribing or use in populations with contraindications to high doses (e.g., elderly, renal impairment).

These variations do not necessarily indicate inappropriate prescribing but highlight the limitations of applying a uniform DDD across all clinical contexts. DDD values are set for the most common indication and average dosing in adults, but NSAIDs are often prescribed for a wide range of conditions, with varying dosing needs. For example, celecoxib may be dosed differently for osteoarthritis versus rheumatoid arthritis. Therefore, calculating DDDs by specific indications could offer a more nuanced and accurate assessment of drug utilization and appropriateness.

### PDDIs

Drug interactions are a major concern for prescribers, especially with the increasing use of multiple drugs to manage complex diseases. Our study found that the overall prevalence of PDDIs in the population was 72.7%, which aligns with other previous studies, reporting prevalence rates ranging from 37.4% to 90.6% (Chaudhari and Chaudhary, 2023; Lule et al., 2024; Mehuys et al., 2022; Hughes et al., 2023). The variation in prevalence rates between studies may stem from differences in the selection of NSAIDs.

Most of the identified drug interactions were classified as minor (61.47%), with only 1.2% classified as significant. These findings contrast with Pappala et al.‘s survey, which found that most interactions (74%) were rated as moderate, and 15.5% were minor (Pappala et al., 2020). Although the majority of identified pDDIs were classified as minor, their clinical impact should not be overlooked. Repeated or chronic exposure to minor interactions—especially in polypharmacy settings—can cumulatively contribute to adverse outcomes such as reduced drug efficacy, increased toxicity, or patient non-adherence. This highlights the need for routine medication reviews even when interactions are deemed low-risk.

In terms of mechanisms, over half of the PDDIs (51.5%) were pharmacodynamic interactions, primarily related to gastrointestinal and renal toxicities. A smaller proportion (27.5%) involved pharmacokinetic interactions. These results are consistent with recent literature on analgesics (Armani et al., 2024). For example, a study in Belgium reported that co-prescribing NSAIDs with antithrombotic agents significantly increased the risk of bleeding and thromboembolism (Mehuys et al., 2022). Similarly, a study in Jordan found that NSAIDs combined with ACE inhibitors, ARBs, diuretics, or antiplatelet agents elevated the risk of nephrotoxicity (Al-Azayzih et al., 2020). These findings emphasize the need for careful prescribing and vigilant monitoring to mitigate the adverse effects associated with NSAID-related DDIs.

In our study, the most common combinations involved NSAIDs with warfarin, escitalopram, spironolactone, clopidogrel, perindopril, gliclazide, and furosemide. These combinations, while sometimes unavoidable due to comorbidities like diabetes or cardiovascular disease, carry increased risks, particularly for gastrointestinal bleeding and renal impairment (Wan et al., 2021). Other drugs commonly co-prescribed with NSAIDs for orthopedic pain and localized swelling include Orphenadrine citrate, glucosamine/chondroitin sulfate, and sodium fascinate.

Additionally, gastroprotective agents, vitamins (such as ascorbic acid), and calcium carbonate were found to interact with NSAIDs, requiring careful patient monitoring for adverse effects. Monitoring for signs or symptoms, laboratory parameters, and assessing the benefit-risk ratio are crucial for ensuring patient safety.

Patients prescribed NSAIDs often take multiple medications to manage comorbidities, which increases the risk of drug interactions and side effects. This can lead to drug-related problems, medication non-compliance, and adverse therapeutic outcomes. To mitigate these risks, it is recommended to utilize a clinical decision support system, which can be highly beneficial for prescribers during the pharmacotherapy of pain-related disorders, ultimately enhancing patient safety (Sutton et al., 2020).

Our study found that the risk of drug interactions significantly increased with factors such as age, gender, nationality, number of prescribed drugs, and comorbidities. Previous literature has highlighted the elderly, polypharmacy, and multiple prescribers as well-established risk factors for potential drug-drug interactions (pDDIs) (Al-Azayzih et al., 2020; Mehuys et al., 2022). A study by Abdu et al. also found that an increase in the number of prescribed drugs and the presence of comorbidities were significantly linked to drug interactions (Abdu et al., 2020). In our study, patients prescribed seven or more medications were at a higher risk of pDDIs compared to those taking fewer than seven.

Our study also observed a significant association between pDDIs and patient nationality. While nationality itself is not a biological determinant of drug interactions, this finding may reflect indirect factors such as variations in prescribing practices, language barriers, and health-seeking behaviors across different national groups. The UAE’s diverse population, with residents from various countries including India, Pakistan, and other Arab nations, may contribute to differences in clinical management approaches and patient-provider communication, which could influence the likelihood of drug interactions.

This finding underscores the need for culturally competent healthcare practices. Providers should be mindful of the demographic makeup of their patient population and consider these factors when prescribing medications. Additionally, further research is necessary to explore whether the observed differences in drug interactions are linked to specific prescribing or documentation practices, as this could provide actionable insights for improving patient safety.

The multivariable logistic regression analysis revealed that the likelihood of PDDIs significantly increased with the number of prescribed medications and the presence of comorbidities (p < 0.001). These findings are consistent with earlier studies linking polypharmacy to an increased risk of PDDIs (Bethi et al., 2018; Gobezie et al., 2021; Aburamadan et al., 2021).

Prescribers must be aware of the risk factors associated with PDDIs. Close and intensive monitoring, ideally through a multidisciplinary approach, can help optimize drug therapy and prevent or minimize PDDIs and associated adverse effects (Bakker et al., 2021). This study highlights the importance of raising awareness about potentially dangerous interactions to improve patient safety.

### Polypharmacy

Polypharmacy was significantly associated with chronic conditions such as diabetes mellitus (OR = 1.446; 95% CI: 1.018–2.054) and cardiovascular disease (OR = 1.818; 95% CI: 1.279–2.585), highlighting the increased likelihood of multiple drug use in these populations. These findings are consistent with earlier studies reporting that individuals with multiple chronic illnesses are more prone to polypharmacy (Doumat et al., 2023; Delara et al., 2022; Alhumaidi et al., 2023). While polypharmacy may be clinically justified in such cases, it increases the risk of potential drug interactions and adverse outcomes. Physicians should take thorough medication histories to avoid unnecessary prescriptions, and pharmacists must educate patients about the risks of self-medication. When polypharmacy is unavoidable, a multidisciplinary approach with close monitoring is essential to ensure safe and effective treatment.

### Clinical implications and recommendations

This study highlights the need for more cautious NSAID prescribing, particularly in high-risk patients with comorbidities or prior gastrointestinal/cardiovascular events. In such cases, using selective COX-2 inhibitors or combining traditional NSAIDs with proton pump inhibitors (PPIs) can reduce the risk of GI complications. However, only 42.18% of patients in our study received PPI co-prescriptions, suggesting a gap in preventive care.

Improved prescriber education and adherence to clinical guidelines are essential. Clinical decision support systems can aid in identifying high-risk scenarios and minimizing potential drug–drug interactions (pDDIs). The strong association between polypharmacy and pDDIs underscores the value of a multidisciplinary approach—engaging physicians, pharmacists, and other healthcare professionals—to ensure safe, personalized, and effective treatment.

### Limitations

This study has several limitations. First, it focused on the Defined Daily Dose (DDD) of overall NSAID consumption, which limited our ability to analyze data regarding the wide variety of indications, doses, and duration of NSAID use. As a result, we were unable to fully quantify drug exposure.

The research was conducted at a single center over a 6-month period, which restricts the generalizability of the findings to the broader prescribing patterns of NSAIDs in the Ras Al Khaimah region. Multi-center studies conducted over longer durations are needed to better understand NSAID usage patterns and associated gastrointestinal (GI) risks.

Additionally, the cross-sectional design and lack of follow-up in this study mean that all recorded drug-drug interactions (DDIs) were theoretical, limiting the assessment of their clinical significance. Incomplete medical records and missing patient data may have also affected our understanding of GI and cardiovascular risks.

While the study focused on clinically relevant pDDIs, it did not consider drugs co-prescribed with NSAIDs that did not result in interactions. Future research should explore this area to gain a more comprehensive understanding of safe co-prescribing patterns, particularly in outpatient settings.

Although we documented the co-prescription of gastroprotective agents with NSAIDs, we did not evaluate whether these prescriptions adhered to clinical guidelines based on patient risk factors. This limits our ability to assess the appropriateness and clinical justification of such co-prescriptions.

Lastly, while we adjusted for key confounding variables based on previous literature, residual or unmeasured confounders may still have influenced the outcomes. Factors such as the severity of illness, patient adherence to medication, or over-the-counter NSAID use were not considered and may affect the generalizability of our findings.

## Conclusion

This study provides valuable insight into NSAID prescribing patterns and associated drug–drug interaction risks in an outpatient setting. While selective COX-2 inhibitors like celecoxib were frequently prescribed due to their gastrointestinal safety profile, they were still involved in clinically relevant pDDIs. Notably, polypharmacy and comorbidities significantly increased the risk of pDDIs, especially in older adults. Furthermore, the low rate of PPI co-prescription (42.18%) highlights a gap in preventive care, particularly for patients at high GI risk. These findings underscore the urgent need for guideline-adherent NSAID prescribing, routine assessment of drug interactions, and the integration of clinical pharmacists in outpatient care to optimize safety and efficacy.

## Data Availability

The data supporting this study is publicly available and can be accessed via the following DOI: https://doi.org/10.6084/m9.figshare.29376935.
